# A Spatiotemporal Analysis of a High-Resolution Molecular Network Reveals Shifts of HIV-1 Transmission Hotspots in Guangzhou, China

**DOI:** 10.3390/v17030384

**Published:** 2025-03-07

**Authors:** Huanchang Yan, Yifan Lu, Shunming Li, Hao Wu, Jingyang Hu, Yefei Luo, Qingmei Li, Lingxuan Lai, Weiping Huang, Jing Gu, Lijun Ma, Yuantao Hao, Zhigang Han, Xin-lin Chen, Yu Liu

**Affiliations:** 1 School of Public Health and Management, Guangzhou University of Chinese Medicine, Guangzhou 510006, China; yanhch5@mail2.sysu.edu.cn (H.Y.); 20221110447@stu.gzucm.edu.cn (Y.L.); kylin1640@yeah.net (L.L.); malj@gzucm.edu.cn (L.M.); 2 Department of Medical Statistics, School of Public Health, Sun Yat-sen University, Guangzhou 510080, China; gujing5@mail.sysu.edu.cn; 3 Department of AIDS Control and Prevention, Guangzhou Center for Disease Control and Prevention, Guangzhou 510440, China; gzcdc_lism@gz.gov.cn (S.L.); gzcdc_wuh@gz.gov.cn (H.W.); yefeiluo@foxmail.com (Y.L.); gzcdc_liqm@gz.gov.cn (Q.L.); 4 School of Medical Information Engineering, Guangzhou University of Chinese Medicine, Guangzhou 510006, China; hujy0505@foxmail.com; 5 Department of Epidemiology, School of Public Health, Southern Medical University, Guangzhou 510515, China; liusu05@foxmail.com; 6 Peking University Center for Public Health and Epidemic Preparedness & Response, Beijing 100191, China; 7 Institute of Public Health, Guangzhou Medical University & Guangzhou Center for Disease Control and Prevention, Guangzhou 510440, China; 8 School of Basic Medical Sciences, Guangzhou University of Chinese Medicine, Guangzhou 510006, China; 9 Guangdong Research Center for TCM Service and Industrial Development, Guangzhou 510006, China

**Keywords:** human immunodeficiency virus, molecular transmission network, spatiotemporal analysis

## Abstract

Background: High-resolution and longitudinal HIV molecular surveillance can inform the evolving hotspots to tailor regionally focused control strategies. Methods: HIV-1 *pol* sequences of three predominant genotypes (CRF01_AE, CRF07_BC, and CRF55_01B) were collected for molecular network reconstruction from people living with HIV (PLWH) in Guangzhou (2018–2020). They were categorized by geographical residences into central, suburban, and outer suburban areas. Clustering rates, assortativity coefficients, and intensity matrices were employed to assess transmission dynamics, geographic mixing patterns, and intra- and inter-area transmission, respectively. Results: Of the 2469 PLWH, 55.5% resided in the central area. Clustering rates showed no significant differences across areas (44.5%, 40.6% vs. 45.7%; *p* = 0.184). However, the transmission hotspots for CRF01_AE and CRF55_01B shifted to the outer suburban area. PLWH tended to form links within their local area (assortativity coefficient = 0.227, *p* < 0.001), particularly for CRF01_AE (0.512, *p* < 0.001; intra-area intensity = 69.2%). The central area exhibited the highest but decreasing intra-area transmission (74.5% to 30.2%), while intra- and inter-area transmission involving the outer suburban area increased (23.1% to 38.2%). Conclusions: Despite most PLWH residing in the central area, the outer suburban area emerged as the hotspot, requiring interventions towards both intra- and inter-area transmission.

## 1. Introduction

The HIV/AIDS epidemic continues to pose a significant global public health challenge, accounting for approximately 88.4 million infections and 42.3 million AIDS-related deaths worldwide [1]. Geographically focused control strategies have emerged as a critical approach to addressing the spatial variations in HIV demographics and prevalence [2,3,4], with increasing evidence demonstrating them as more cost-effective than universal approaches in reducing HIV transmission [5]. Therefore, continuous and deep sampling coupled with high geo-resolution analysis is essential for designing precise regional HIV interventions that adapt to the evolving hotspots of transmission.

Advances in sequencing and analytical methods have enabled the detection of potential transmission among people living with HIV (PLWH) through HIV molecular transmission networks [6]. In these networks, an inferred HIV transmission link is assigned to any two PLWH whose viral sequences exhibit high similarity. Integrating molecular network analysis into surveillance toolkits helps overcome challenges posed by geographic differences [7], which are closely associated with HIV transmission [8]. Spatialized HIV molecular transmission networks have been shown to unveil spatial heterogeneities in HIV transmission, enhance understanding of the evolving dynamics of HIV, and provide critical insights into geo-targeted control at a regional scale [3,9,10]. However, previous regional HIV molecular networks have focused on cross-sectional rather than longitudinal surveillance data, limiting their ability to capture dynamic shifts in geographically dispersed transmission patterns over time.

Recently, the HIV molecular network has been spatialized to illuminate both intra- and inter-regional transmission connections [3], representing a significant advancement over traditional approaches that merely describe the geographical distribution of prevalent genotypes [11,12,13]. In this study, we dynamically spatialized the HIV molecular network in Guangzhou, a megacity in China with a population of 18.83 million from various regions of the country. Over 80% of reported HIV infections in this city are associated with three primary HIV-1 genotypes: CRF07_BC, CRF01_AE, and CRF55_01B [14]. The geographical dissemination of these genotypes is presumably driven by economic vitality and transportation accessibility. Thus, we categorized the residences of PLWH into distinct sub-areas according to their geo-distance from the city center. This study revealed the spatiotemporal dynamics of HIV transmission hotspots within these areas, aiming to provide precise information that can guide the development of tailored prevention and control strategies, optimize the allocation of health resources, and effectively contain the HIV epidemic.

## 2. Material and Methods

### 2.1. Study Design

This study retrospectively enrolled treatment-naïve Chinese adults who were newly diagnosed with HIV/AIDS in Guangzhou during 2018–2020. Following standardized procedures outlined in our previous research [14], clinical specimens were collected, and HIV-1 DNA sequencing and subtyping were performed, resulting in a dataset of 3176 cases with their corresponding pol gene sequences (HXB2: 2253-3821nt). Subsequently, the demographic, social, and clinical characteristics of these cases were extracted from the China Information Management System for Comprehensive Prevention and Control of AIDS, including age at diagnosis, gender, marital status, education, residence, self-reported HIV infection, total number of reported contacts through sexual behavior or sharing needles, HIV status at first diagnosis, and status of follow-up.

**Figure 1:**
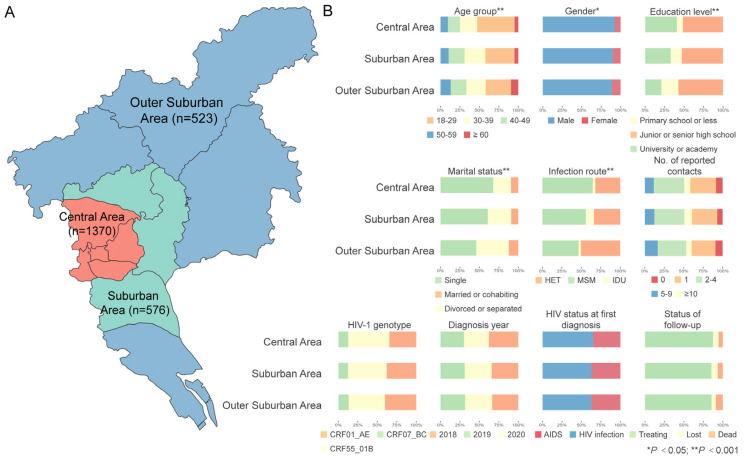
Characteristics of people living with HIV (PLWH) diagnosed with the three main genotypes in Guangzhou, China (2018–2020). (**A**) Distribution of PLWH across three areas: central, suburban, and outer suburban. (**B**) Demographic and social characteristics of PLWH in each area.

Subsequent data curation involved applying dual exclusion criteria: (1) elimination of non-target genotypes and (2) PLWH residing outside Guangzhou. This refined the analytical cohort to 2469 cases and their pol gene sequences representing the three prevalent genotypes (i.e., CRF01_AE, CRF07_BC, and CRF55_01B) in Guangzhou. According to the residence of PLWH, these sequences were classified by their geographic origins into central (Yuexiu, Liwan, Tianhe, Haizhu, and Baiyun south of the North Second Ring Expressway), suburban (Baiyun north of the North Second Ring Expressway, Huangpu, Panyu), and outer suburban (Nansha, Zengcheng, Huadu, Conghua) areas of Guangzhou (Figure 1).

This study was approved by the Institution Review Board of the School of Public Health, Sun Yat-sen University (No. 2023073). All data were anonymized prior to analysis.

### 2.2. Molecular Network Analysis

HIV-TRACE was used to construct molecular networks of CRF01_AE, CRF07_BC, and CRF55_01B, and the Tamura-Nei 93 (TN93) nucleotide substitution model was employed to calculate the pairwise genetic distance (GD). To investigate the optimal threshold of GD for molecular network construction, we applied a sensitivity analysis spanning a spectrum of GD thresholds, ranging from 0.001 substitutions/site to 0.015 substitutions/site. The optimal GD threshold was identified as the minimum threshold that detected the maximum total number of clusters (including pairs) in the molecular networks [6]. PLWH were represented by nodes, and nodes were linked when their pairwise GD was equal to or lower than the optimal threshold. The molecular network was visualized using Cytoscape 3.10.2 [15]. Clustering rates were calculated for each HIV-1 genotype to reflect the transmission dynamics in each area of Guangzhou. The clustering rate was defined as the proportion of the PLWH clustered in the molecular network over all PLWH in a given area during a given year. A higher clustering rate demonstrates more genetic relatedness of local HIV-1 transmission.

HIV-1 molecular networks were further spatialized to identify the hotspots of genotype-specific transmission to reflect HIV transmission geographically. The proportion of intra-area transmission during each year was calculated by dividing the number of links between PLWH in the area by the number of links with any PLWH in the area, while the remaining proportion was referred to as the proportion of inter-area transmission [3]. Each link was assigned to the latter year that the two linked PLWH were diagnosed. Additionally, the transmission intensity was visualized and colored in the matrices. The color of the grid cell at the intersection of two areas represented the number of links between the PLWH in these two areas.

### 2.3. Geographic Assortativity

Assortativity in a network refers to the tendency of those with similar attributes to preferentially connect [16]. To describe spatial mixing patterns in both the overall and genotype-specific molecular networks, assortativity coefficients were further calculated. The assortativity coefficient ranges from −1 to 1, where negative values represent a preference for links between PLWH residing in two different areas, and positive values represent a preference for connection between PLWH in the same area; zero value represents random mixing with no tendency for assortativity. Significance for geographic assortativity was determined using 10,000 random permutations of the molecular transmission network.

### 2.4. Statistical Analysis

The clustering rates between areas were compared using χ^2^ tests. Univariate and multivariable logistic regression models were used to identify factors associated with clustering, while the outcome was defined as either clustered in the network (1) or not (0). For clustered PLWH, logistic regression models were further applied to determine factors associated with inter-area transmission, with the outcome defined as either having any links (1) or not (0). Demographic, social, and clinical characteristics were included as predictor variables. *p* ≤ 0.05 was considered statistically significant. The analyses were conducted in R v4.2.3.

## 3. Results

### 3.1. Characteristics of the Study Population

In total, 3176 pol gene sequences were collected from Chinese adults newly diagnosed between 2018 and 2020 in Guangzhou, China. After removing sequences of other HIV-1 genotypes and PLWH living outside Guangzhou, a total of 2469 HIV-1 pol gene sequences of HIV-1 CRF01_AE (903, 36.6%), CRF07_BC (1255, 50.8%), and CRF55_01B (311, 12.6%) were collected from PLWH newly diagnosed in Guangzhou between 2018 and 2020. These PLWH were primarily diagnosed at the age of 18–29 (42.4%), male (91.0%), single (61.4%), and had a junior or senior high school education (53.2%). Most of the PLWH were infected through men who have sex with men (MSM, 58.7%), reported 2–4 close contacts (38.4%), were diagnosed at the HIV infection stage (64.1%), and were under treatment (86.6%).

The central area of Guangzhou was the residence for over half of these PLWH (1370, 55.5%), followed by the suburban (576, 23.3%) and outer suburban (523, 21.2%) areas (Figure 1A). A significant difference was observed among PLWH residing in the central, suburban, and outer suburban areas with regard to age at diagnosis (*p* < 0.001), gender (*p* = 0.041), education (*p* < 0.001), marital status (*p* < 0.001) and infection route (*p* < 0.001) (Figure 1B). Of note, most of the PLWH in the central area were young at diagnosis (48.2%), male (92.3%), single (67.6%), infected through MSM (64.7%), and had high school or above education (92.4%).

### 3.2. Spatiotemporal Patterns of Clustering Rates

**Figure 2:**
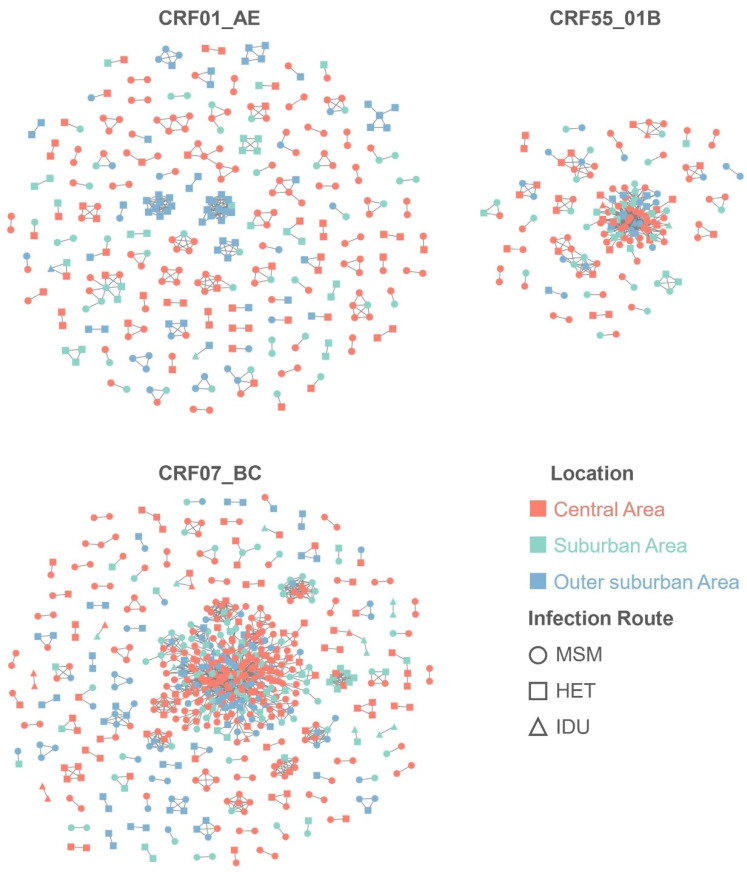
Molecular networks of CRF01_AE, CRF07_BC, and CRF55_01B in Guangzhou, China. Molecular networks were constructed using a pairwise genetic distance threshold of 0.012 substitutions per site. Rows represent the genotypes (CRF01_AE, CRF07_BC, and CRF55_01B) and columns display data for each year (2018, 2019, and 2020). Colors represent different areas of Guangzhou, while shapes indicate infection routes. HET, heterosexual transmission; IDU, injecting drug users; MSM, men who have sex with men.

Using the optimal GD threshold of 0.012 substitutions/site (Figure S1), we constructed molecular networks comprising 1082 sequences distributed in 253 clusters (Figure 2). The size of these clusters exhibited considerable heterogeneity; notably, the two largest clusters included 297 sequences of the CRF07_BC strain and 71 sequences of the CRF55_01B strain, while 163 clusters consisted of only two sequences each. The overall clustering rates for the three HIV-1 genotypes were significantly different (*p* < 0.001), with rates of 35.6% for CRF01_AE, 48.0% for CRF07_BC, and 50.8% for CRF55_01B. Logistic regression analysis showed that PLWH were more likely to be clustered if they were ≥60, male, infected through MSM, diagnosed during 2018, and infected by CRF07_BC and CRF55_01B (Table S1).

**Figure 3:**
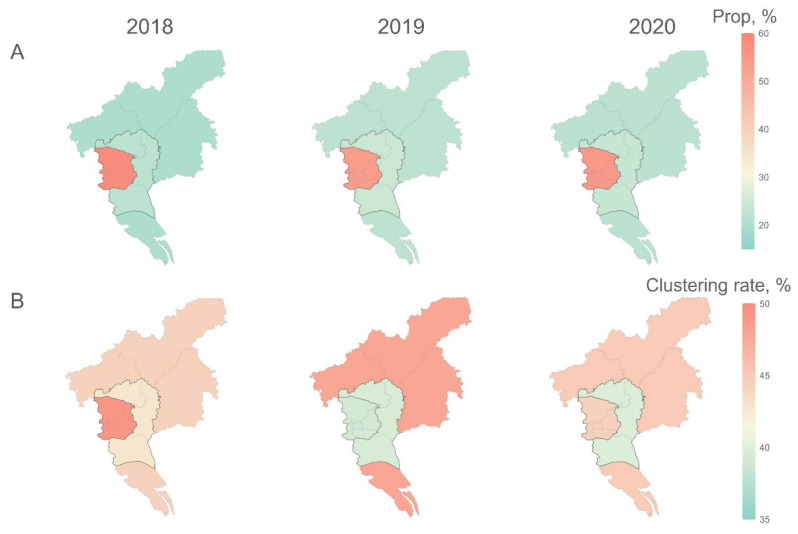
Spatiotemporal distribution of people living with HIV (**A**) and clustering rates (**B**) in Guangzhou from 2018 to 2020. Maps of PLWH are colored by the proportions of PLWH in a given year, while those of clustering rates are colored by absolute values. Higher values are represented by red hues, whereas lower values are represented in green.

Though most of the PLWH resided in urban areas annually, the clustering rates among PLWH in the three areas were 44.5%, 40.6%, and 45.7%, respectively, with no statistically significant differences (*p* = 0.186) (Figure 3; Table S2); similar results were observed for each genotype (Figure S2). Notably, the spatiotemporal distribution of clustering rates varied by genotype: in 2019, the hotspots for overall and CRF01_AE clustering rates shifted from the urban area to the outer suburban area, and the CRF55_01B hotspot shifted from the suburban area to the outer suburban area; the CRF07_BC hotspot shifted from the outer suburban area to the central area in 2020. Specifically, in the CRF01_AE networks, the highest clustering rate was observed in the central area in 2018 (43.1%, *p* < 0.001), shifting to the outer suburban area in 2019 (42.3%, *p* = 0.162) and 2020 (40.6%, *p* = 0.343). In contrast, the highest clustering rate of CRF07_BC was observed in the outer suburban area in 2018 (57.7%, *p* < 0.001) and 2019 (57.5%, *p* = 0.007), moving to the central area in 2020 (50.9%, *p* = 0.772). Notably, the CRF55_01B hotspot shifted from the suburban area in 2018 (69.6%, *p* = 0.314) to the central area in 2019 (62.2%, *p* = 0.032), and then back to the suburban area in 2020 (48.0%, *p* = 0.845).

### 3.3. Intra-Area and Inter-Area Transmission Links

The assortativity coefficient of 0.227 (*p* < 0.001) revealed a significant tendency for PLWH to form potential transmission links with others in the same area (Table 1). Notably, significant positive assortativity coefficients were observed within the network of each HIV-1 genotype, with CRF01_AE exhibiting the highest assortativity of 0.512 (*p* < 0.001) and CRF55_01B the lowest at 0.060 (*p* < 0.001). Particularly high assortativity coefficients were found among PLWH residing in the outer suburban (0.693, *p* < 0.001) and central (0.501, *p* < 0.001) areas in the CRF01_AE molecular network, whereas the CRF55_01B network was less assortative (assortativity coefficients < 0.1) than the other networks.

**Figure 4:**
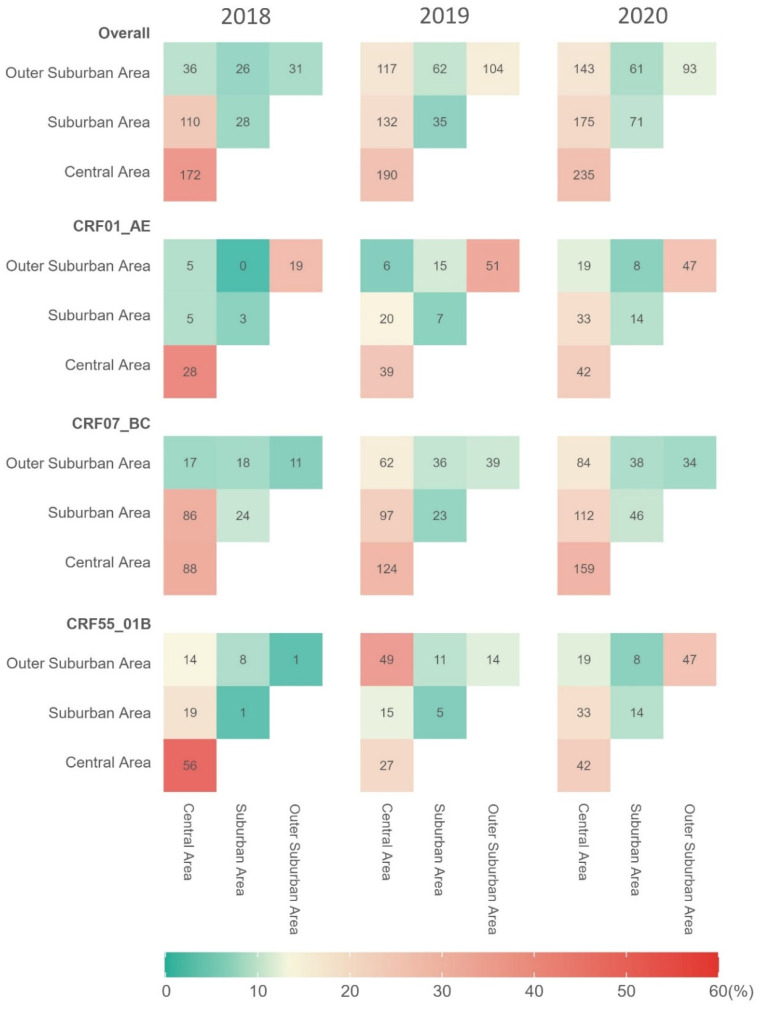
Spatiotemporal distribution of HIV-1 transmission intensities across different areas of Guangzhou from 2018 to 2020. Colors indicate the proportions of transmission intensity in a given year, with red hues representing higher proportions and green hues representing lower proportions. The color of each grid cell at the intersection of two areas indicates the number of links between the people living with HIV in two areas.

Out of 1821 potential transmission links, over half were detected between PLWH residing in the same areas (959/1821, 52.7%; Table S3). Approximately 70% of CRF01_AE links (69.3%) were intra-area transmission, while this proportion was less than half of CRF07_BC (49.9%) or CRF55_01B links (44.5%). The proportion of intra-area transmission in the CRF01_AE molecular network decreased from 83.3% in 2018 to 63.2% in 2020, while the lowest proportion of intra-area transmission was observed in the CRF55_01B molecular network in 2019 (46/121, 38.0%). The central area exhibited the highest intra-area transmission intensity, with a decrease in the proportion of these links over time (from 74.5% to 30.2%; Figure 4). In contrast, the outer suburban area showed a growth in the proportion of intra- and inter-area transmission (from 23.1% to 38.2%). PLWH in the suburban area had a relatively small number (71) of intra-area transmission links across HIV-1 genotypes, whereas intra-area transmission of CRF01_AE and CRF55_01B increased within the outer suburban area.

Furthermore, transmission matrices showed distinct spatial heterogeneity in HIV transmission. Over time, HIV transmission intensity increased, with the central area consistently showing the highest inter-area transmission intensity (713/862, 82.7%; Figure 4). In 2018, inter-area transmissions were relatively low between the central and outer suburban areas in 2018, while sharp increases were observed between the central and the other areas in 2019, particularly for CRF07_BC and CRF55_01B (Table S3). During 2020, inter-area links between the central area and outer suburban area peaked across HIV-1 genotypes, suggesting a potential shift of transmission hotspots from the urban core to more peripheral areas. Notably, CRF01_AE transmission matrices showed a large rise in inter-area links between the central and outer suburban areas, while CRF07_BC and CRF55_01B maintained stronger connections between the central and suburban areas.

### 3.4. Factors Associated with Inter-Area Transmission

Univariate and multivariate logistic regression analyses were used to identify factors associated with inter-area transmission among the clustered PLWH (Table S4). The multivariate analysis showed that male PLWH were around 4-fold more likely to form inter-area transmission links than female PLWH (OR: 3.681, 95% CI: 1.911–7.300). In addition, PLWH residing in the suburban (OR: 2.446, 95% CI: 1.682–3.605) and outer suburban (OR: 2.103, 95% CI: 1.443–3.097) areas tended to have inter-area transmission, whereas PLWH infected by CRF07_BC (OR: 1.970, 95% CI: 1.445–2.692) and CRF55_01B (OR: 3.529, 95% CI: 2.193–5.798) were more likely to be involved in inter-area transmission than those infected by CRF01_AE.

## 4. Discussion

Using longitudinal surveillance data, we characterized the spatiotemporal dynamics of HIV transmission across Guangzhou and identified the shifts in hotspots. While the majority of PLWH newly diagnosed in Guangzhou between 2008 and 2020 resided in the central area, the spatiotemporal distribution of genotype-specific transmissions indicated a diminishing role of the central area as a hotspot and a growing importance of the outer suburban area. Our findings indicate a tendency for PLWH to form transmission links within their own areas, particularly within the CRF01_AE network. In contrast, the CRF07_BC and CRF55_01B networks displayed lower assortativity coefficients and extensive inter-area transmission links, underscoring the complexity of their spread.

The central areas with the highest number of PLWH may not align with priority intervention areas in Guangzhou. In the urban setting, clustering in HIV molecular networks is an essential measure to understand spatial transmission dynamics and guide precision prevention strategies [17]. Though newly diagnosed PLWH in Guangzhou were predominantly located in the central area, our analysis revealed shifting genotype-specific hotspots. In 2018, the highest clustering rates for overall cases, CRF01_AE, and CRF07_BC were observed in the central area. However, this trend declined over time, indicating that central urban residents were less likely to contribute to local transmission chains, and a large proportion of non-clustered PLWH might be related to transmission outside Guangzhou [18]. Transmission matrices showed similar patterns that the intra-area transmission intensity within the central area initially dominated local HIV-1 transmission but then decreased in proportion. These findings emphasized the critical need to target prevention efforts at regional hotspots rather than merely focusing on areas with high prevalence.

Notably, hotspots for CRF01_AE and CRF07_BC shifted to the outer suburban area after 2018, marking it as an emerging hotspot. After 2018, increasing intra-area transmissions of CRF01_AE and CRF55_01B were also observed within the outer suburban area. Compared with the clustered PLWH in the central area, those in the outer suburban and suburban areas were more likely to form inter-area transmission in Guangzhou. These shifts may be attributed to the expansion of the Guangzhou Metro into outer suburban areas through new lines (Lines 4, 9, 13, 14, and 21) from late 2017 to 2020. These additions enhanced connectivity between the central area and the outer suburban area, facilitating cross-area movement. However, public transportation in the vast outer suburban area remains less developed than in the central area, making it more vulnerable to localized outbreaks if a virus is introduced. The outer suburban area, in particular, requires enhanced surveillance and interventions to address its growing role in transmission.

Distinct transmission patterns among genotypes provided further insights into the dynamic of HIV-1 spread. The CRF01_AE molecular network exhibited strong assortativity by residence, especially in the central and outer suburban areas, which contrasted with patterns observed in Los Angeles County, USA [17], and Cologne-Bonn, Germany [19]. These findings suggested geographically localized prevention strategies, such as community-based health education programs. In contrast, CRF55_01B demonstrated the lowest assortativity by residence but the strongest inter-area transmission intensity. Notably, clustered PLWH infected by CRF55_01B were 2.5-fold more likely to form inter-area links than CRF01_AE. This pattern might be influenced by factors such as the well-developed public transportation network and the mobility of the young MSM population, who were disproportionately affected by the CRF55_01B epidemic (accounted for 65.3% of CRF55_01B population in our study) [20] and frequently seek sex partners via the Internet [21,22]. These findings underscore the importance of cross-area collaboration involving contact tracing among public health departments and targeted interventions leveraging digital platforms.

There were several limitations of our study. First, despite achieving a relatively high level of sequence reporting completeness in HIV molecular surveillance in Guangzhou [14], missing nodes and links in molecular networks may arise from undetected PLWH or sequencing failure. Thus, it is recommended that molecular surveillance capabilities be enhanced. Second, the high population mobility interferes with the detection of transmission hotspots. For precise prevention and control based on the spread of hotspots, near real-time monitoring may be more effective. Additionally, clustering rates and transmission intensity matrices may vary with different GD thresholds; however, we applied a single optimal threshold based on prior research on spatial patterns [3,10,23]. Finally, the molecular networks reconstructed from consensus sequences allow for the identification of potential transmission links but do not indicate transmission direction. Further analysis using deep-sequencing data could offer valuable insights into the spatiotemporal transmission patterns, including transmission direction [24].

## 5. Conclusions

Although over half of the PLWH newly diagnosed in Guangzhou resided in the central area, the outer suburban area emerged as the hotspot for local HIV-1 transmission in Guangzhou. Our findings underscore the urgent need for enhanced surveillance within these hotspots and strengthened inter-area collaboration to effectively curb local transmission.

## Figures and Tables

**Table 1:** Assortativity of the Guangzhou molecular networks of CRF01_AE, CRF07_BC, and CRF55_01B by residence. Significance for geographic assortativity was determined using 10,000 random permutations of the molecular transmission network.

Area	Overall		CRF01_AE		CRF07_BC		CRF55_01B	
	Assortativity	*p*	Assortativity	*p*	Assortativity	*p*	Assortativity	*p*
Guangzhou	0.227	<0.001	0.512	<0.001	0.160	<0.001	0.060	<0.001
Central area	0.215	<0.001	0.501	<0.001	0.158	<0.001	0.067	0.038
Suburban area	0.120	<0.001	0.235	<0.001	0.086	0.002	0.092	0.017
Outer suburban area	0.344	<0.001	0.693	<0.001	0.253	<0.001	0.026	0.174

## Data Availability

The data that support the findings of this study are available on request from corresponding authors. The data are not publicly available due to privacy or ethical restrictions.

## Supplementary Materials

The following supporting information can be downloaded at: https://www.mdpi.com/article/10.3390/v17030384/s1, Figure S1: Number of clusters across pairwise genetic distance (GD) thresholds ranged from 0.001 to 0.015 substitutions/site; Figure S2: Spatiotemporal distribution of genotype-specific people living with HIV(PLWH) and clustering rates in Guangzhou from 2018 to 2020; Table S1: Associations of each characteristic with being within HIV-1 molecular clusters using bivariate and multivariate logistic regressions; Table S2: Spatiotemporal clustering rates across HIV-1 genotypes in Guangzhou, China; Table S3: Spatiotemporal patterns of HIV-1 intra-area and inter-area transmission proportions across genotypes in Guangzhou, China; Table S4: Associations of each characteristic with having inter-area transmission using bivariate and multivariate logistic regressions.
