# Supplementary material for: A Spatiotemporal Analysis of a High-Resolution Molecular Network Reveals Shifts of HIV-1 Transmission Hotspots in Guangzhou, China
Source: Viruses. 2025 Mar 7;17(3):384. doi: 10.3390/v17030384 (PMC11945462; doi:10.3390/v17030384)
Supplement: Supplementary file 1 [file viruses-17-00384-s001.zip › viruses-3460911-supplementary.pdf]

Supplementary Materials

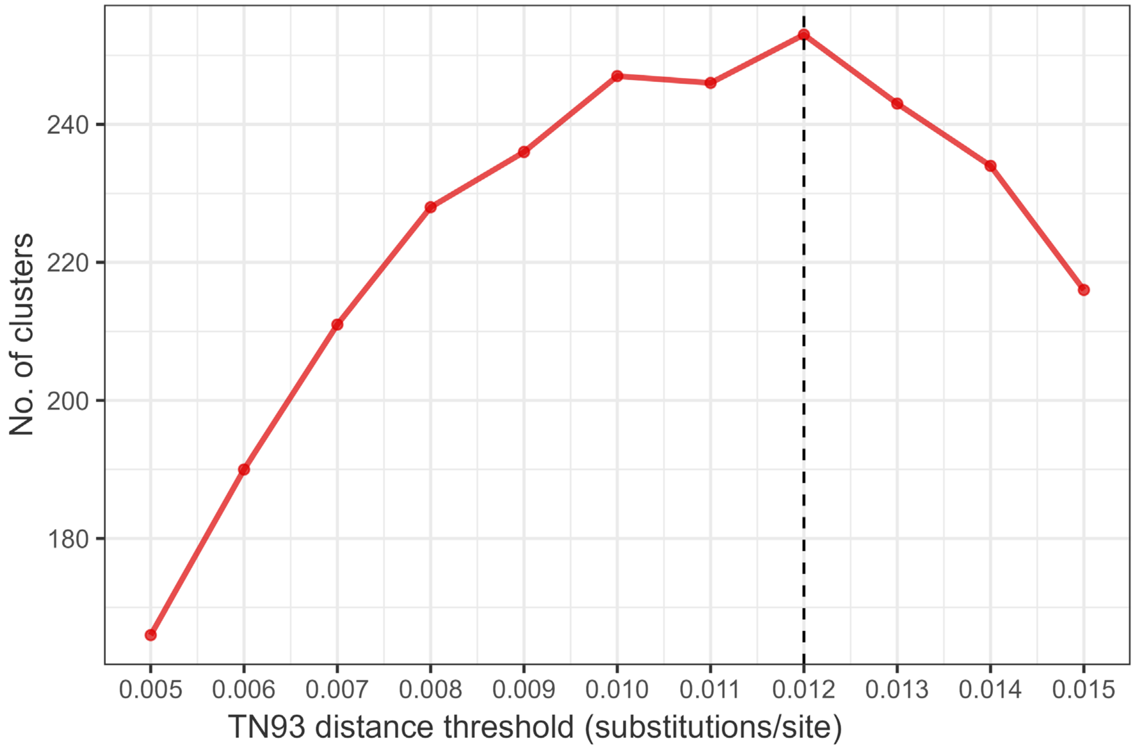

Figure S1 Number of clusters across pairwise genetic distance (GD) thresholds ranged from 0.001 to 0.015 substitutions/site. The optimal GD threshold was determined as 0.012 substitutions/site, which identified the highest number of clusters in the molecular networks.

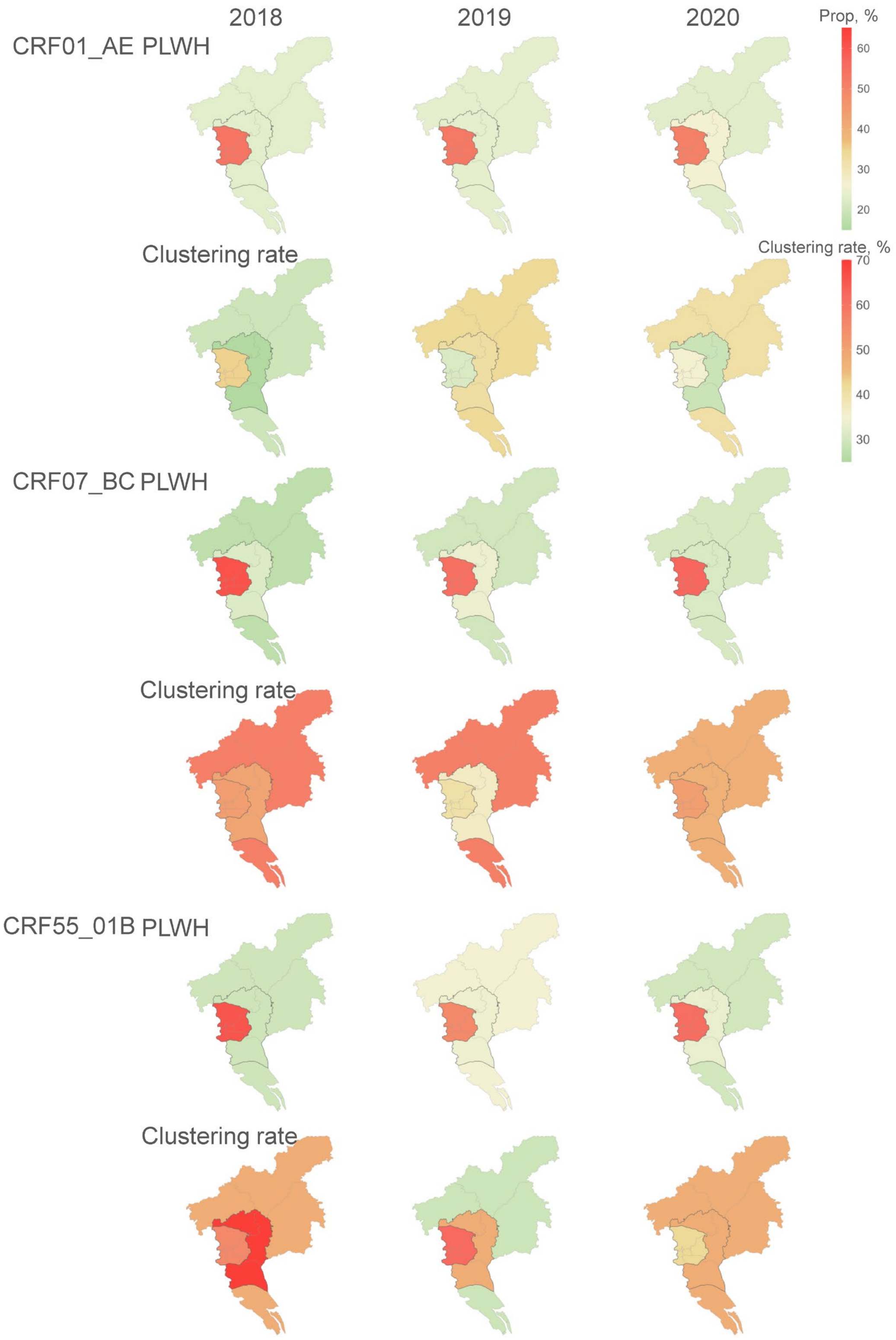

Figure S2 Spatiotemporal distribution of genotype-specific people living with HIV(PLWH) and clustering rates in Guangzhou from 2018 to 2020. Maps of PLWH are colored by the proportions of PLWH in a given year while those of clustering rates by absolute values.

Table S1 Associations of each characteristic with being within HIV-1 molecular clusters using bivariate and multivariate logistic regressions.

Characteristic	Clustered (n=1082)	unadjusted OR (95% CI)	adjusted OR (95% CI)
**Age group**			
≥60	77 (54.23)	1	1
**18-29**	**496 (47.37)**	**0.760 (0.533-1.079)**	**0.527 (0.321-0.862)**
**30-39**	**230 (39.38)**	**0.548 (0.378-0.793)**	**0.422 (0.263-0.675)**
**40-49**	**180 (41.28)**	**0.594 (0.405-0.868)**	**0.582 (0.374-0.903)**
**50-59**	**99 (38.08)**	**0.519 (0.342-0.784)**	**0.533 (0.334-0.847)**
**Gender**			
Female	71 (32.13)	1	1
Male	1011 (44.97)	1.727 (1.292-2.329)	1.416 (0.964-2.097)
**Education level**			
Junior or high school	570 (43.51)	1	1
Primary school or less	119 (39.67)	0.854 (0.660-1.101)	0.956 (0.683-1.335)
University or academy	392 (45.90)	1.102 (0.926-1.310)	0.944 (0.767-1.162)
**Marital status**			
Divorce or separated	98 (39.84)	1	1
Married or cohabiting	294 (41.64)	1.078 (0.803-1.451)	0.986 (0.698-1.395)
Single	689 (45.45)	1.258 (0.958-1.659)	1.138 (0.769-1.687)
**Location of Residence**			
Central area	609 (44.45)	1	1
Suburban area	234 (41.49)	0.855 (0.701-1.041)	1.013 (0.809-1.267)
Outer suburban area	239 (44.74)	1.052 (0.859-1.287)	1.207 (0.955-1.526)
**Infection route**			
HET	361 (40.11)	1	1
IDU	28 (13.53)	0.459 (0.290-0.707)	0.332 (0.145-0.692)
MSM	693 (47.79)	1.367 (1.155-1.618)	1.284 (1.021-1.615)
**No. of reported contacts**			
0	90 (44.33)	1	1
1	349 (44.12)	0.991 (0.727-1.354)	0.817 (0.568-1.175)
2~4	407 (43.44)	0.964 (0.711-1.311)	0.734 (0.507-1.062)
5~9	147 (47.12)	1.119 (0.785-1.597)	0.830 (0.542-1.270)
≥10	81 (40.70)	0.862 (0.580-1.280)	0.646 (0.405-1.026)
**Diagnosis year**			
2018	416 (46.69)	1	1
**2019**	**338 (41.17)**	**0.799 (0.660-0.967)**	**0.708 (0.573-0.874)**
**2020**	**328 (43.33)**	**0.873 (0.718-1.061)**	**0.798 (0.642-0.991)**
**HIV status at first diagnosis**			
AIDS	347 (40.68)	1	1
**HIV infection**	**700 (46.05)**	**1.245 (1.051-1.476)**	**1.153 (0.951-1.397)**

Continued to Table S1

Characteristic	Clustered (n=1082)	unadjusted OR (95% CI)	adjusted OR (95% CI)
**Status of follow-up**			
Dead	52 (38.24)	1	1
Drop	65 (43.33)	1.235 (0.770-1.987)	1.265 (0.750-2.137)
Treating	852 (46.13)	1.383 (0.971-1.988)	1.265 (0.857-1.880)
**HIV-1 genotype**			
CRF01_AE	321 (35.59)	1	1
**CRF07_BC**	**603 (48.05)**	**1.677 (1.407-2.000)**	**1.721 (1.416-2.093)**
**CRF55_01B**	**158 (50.80)**	**1.872 (1.443-2.431)**	**1.891 (1.421-2.520)**

OR, odds ratio; CI, confidence interval; HET, heterosexual transmission; IDU, injecting drug use; MSM, men who have sex with men.

Table S2 Spatiotemporal clustering rates across HIV-1 genotypes in Guangzhou, China.

Location of Residence	2018		2019		2020		Overall	
	Clustering rate (%)	*P*	Clustering rate (%)	*P*	Clustering rate (%)	*P*	Clustering rate (%)	*P*
Overall		0.242		0.119		0.523		0.186
Central area	49.0		39.2		44.3		44.5	
Suburban area	42.6		39.5		39.7		40.6	
Outer suburban area	44.3		47.8		44.8		45.7	
CRF01_AE		**0.011**		0.162		0.343		0.355
Central area	**43.10**		31.01		35.21		36.71	
Suburban area	**25.33**		40.85		28.77		31.51	
Outer suburban area	**29.33**		42.25		40.63		37.14	
CRF07_BC		**<0.001**		**0.007**		0.772		0.057
Central area	**51.09**		**40.25**		50.93		47.51	
Suburban area	**49.49**		**36.79**		46.91		44.06	
Outer suburban area	**57.69**		**57.47**		47.50		54.29	
CRF55_01B		0.314		**0.032**		0.845		0.191
Central area	55.71		**62.22**		42.11		52.91	
Suburban area	69.57		**47.83**		48.00		54.93	
Outer suburban area	47.83		**29.17**		47.62		41.18	

Table S3 Spatiotemporal patterns of HIV-1 intra-area and inter-area transmission proportions across genotypes in Guangzhou, China.

Area	2018		2019		2020		Total	
	Intra-area	Inter-area	Intra-area	Inter-area	Intra-area	Inter-area	Intra-area	Inter-area
Overall	57.32	42.68	51.41	48.59	51.29	48.71	52.66	47.34
Central area	54.09	45.91	43.18	56.82	42.50	57.50	**45.57**	54.43
Suburban area	17.07	82.93	15.22	84.78	23.13	76.87	19.14	80.86
Outer suburban area	33.33	66.67	36.75	63.25	31.31	68.69	33.88	66.12
CRF01_AE	83.33	16.67	70.29	29.71	63.19	36.81	69.25	30.75
Central area	73.68	26.32	60.00	40.00	44.68	55.32	**55.33**	44.67
Suburban area	37.50	62.50	16.67	83.33	25.45	74.55	22.86	77.14
Outer suburban area	79.17	20.83	70.83	29.17	63.51	36.49	**68.82**	31.18
CRF07_BC	50.41	49.59	48.82	51.18	50.53	49.47	49.91	50.09
Central area	46.07	53.93	43.66	56.34	44.79	55.21	**44.75**	55.25
Suburban area	18.75	81.25	14.65	85.35	23.47	76.53	19.38	80.62
Outer suburban area	23.91	76.09	28.47	71.53	21.79	78.21	24.78	75.22
CRF55_01B	58.59	41.41	38.02	61.98	40.14	59.86	44.48	55.52
Central area	62.92	37.08	29.67	70.33	32.69	67.31	**41.20**	58.80
Suburban area	3.57	96.43	16.13	83.87	19.64	80.36	14.78	85.22
Outer suburban area	4.35	95.65	18.92	81.08	17.91	82.09	16.46	83.54

Table S4 Associations of each characteristic with having inter-area transmission using bivariate and multivariate logistic regressions.

Characteristic	Having inter-area links (n=672)	unadjusted OR (95% CI)	adjusted OR (95% CI)
**Age group**			
≥60	34 (44.16)	1	1
18-29	323 (65.12)	2.361 (1.455-3.860)	1.506 (0.697-3.260)
30-39	141 (61.3)	2.004 (1.191-3.394)	1.292 (0.620-2.702)
40-49	111 (61.67)	2.035 (1.188-3.512)	1.444 (0.748-2.802)
50-59	63 (63.64)	2.213 (1.210-4.094)	1.922 (0.919-4.067)
**Gender**			
Female	23 (32.39)	1	1
**Male**	**649 (64.19)**	**3.742 (2.265-6.354)**	**3.681 (1.911-7.300)**
**Education level**			
Junior or high school	363 (63.68)	1	1
Primary school or less	55 (46.22)	0.490 (0.328-0.730)	0.687 (0.393-1.203)
University or academy	253 (64.54)	1.038 (0.794-1.358)	1.040 (0.750-1.443)
**Marital status**			
Divorce or separated	62 (63.27)	1	1
Married or cohabiting	169 (57.48)	0.785 (0.487-1.252)	0.828 (0.463-1.462)
Single	441 (64.01)	1.033 (0.660-1.594)	0.881 (0.451-1.698)
**Location of Residence**			
Central area	345 (56.65)	1	1
**Suburban area**	**171 (73.08)**	**2.077 (1.499-2.905)**	**2.446 (1.682-3.605)**
**Outer suburban area**	**156 (65.27)**	**1.438 (1.056-1.968)**	**2.103 (1.443-3.097)**
**Infection route**			
HET	199 (55.12)	1	1
IDU	17 (60.71)	1.258 (0.579-2.840)	2.806 (0.572-21.479)
MSM	456 (65.8)	1.566 (1.207-2.032)	1.129 (0.777-1.636)
**No. of reported contacts**			
0	43 (47.78)	1	1
1	224 (64.18)	1.959 (1.227-3.135)	1.206 (0.685-2.107)
2~4	254 (62.41)	1.815 (1.146-2.880)	0.917 (0.515-1.618)
5~9	96 (65.31)	2.057 (1.207-3.528)	0.929 (0.477-1.800)
≥10	51 (62.96)	1.858 (1.012-3.449)	0.975 (0.466-2.042)
**Diagnosis year**			
2018	273 (65.63)	1	1
2019	201 (59.47)	0.769 (0.571-1.034)	0.768 (0.549-1.075)
2020	198 (60.37)	0.798 (0.591-1.077)	0.839 (0.597-1.178)
**HIV status at first diagnosis**			
AIDS	215 (62.96)	1	1
HIV infection	437 (62.43)	1.020 (0.782-1.329)	0.994 (0.730-1.352)

Continued to Table S4

Characteristic	Having inter-area links (n=672)	unadjusted OR (95% CI)	adjusted OR (95% CI)
**Status of follow-up**			
Dead	31 (59.62)	1	1
Drop	43 (66.15)	1.324 (0.621-2.830)	1.283 (0.531-3.131)
Treating	527 (61.85)	1.098 (0.613-1.933)	0.977 (0.498-1.882)
**HIV-1 genotype**			
CRF01_AE	159 (49.53)	1	1
**CRF07_BC**	**390 (64.68)**	**1.866 (1.417-2.458)**	**1.970 (1.445-2.692)**
**CRF55_01B**	**123 (77.85)**	**3.581 (2.339-5.590)**	**3.529 (2.193-5.798)**

OR, odds ratio; CI, confidence interval; HET, heterosexual transmission; IDU, injecting drug use; MSM, men who have sex with men.
